# Allicin protects against renal ischemia–reperfusion injury by attenuating oxidative stress and apoptosis

**DOI:** 10.1007/s11255-021-03014-2

**Published:** 2021-11-25

**Authors:** Maolin Li, Jinzhuo Ning, Houbao Huang, Shuchuan Jiang, Dong Zhuo

**Affiliations:** 1 Department of Urology, Wuxi People’s Hospital of Nanjing Medical University, Wuxi, Jiangsu Province People’s Republic of China; 2 Department of Urology, Renmin Hospital of Wuhan University, Wuhan, Hubei Province 430060 People’s Republic of China; 3 Department of Urology, The First Affiliated Hospital of Wannan Medical College, Wuhu, Anhui Province 241002 People’s Republic of China

**Keywords:** Allicin, Renal ischemia–reperfusion injury, Reactive Oxygen Species, Apoptosis

## Abstract

**Background:**

Studies have demonstrated that allicin may play critical roles in the procession of ischemia–reperfusion(I/R) injury. The purpose of this study was to investigate the protective effects of allicin on renal I/R injury by attenuating oxidative stress and apoptosis.

**Methods:**

To establish a model of renal I/R, the right kidney underwent 12 h reperfusion after 45 min ischemia, allicin was administered intraperitoneally at concentrations of 40, 50 or 60 mg/kg. NRK-52E cells were treated with allicin at concentrations of 1, 3 or 5 μM in 24 h hypoxia/ 6 h reoxygenation(H/R) treatments. Indicators of HE, oxidative stress, apoptosis were measured to evaluate the effect of aliicin on renal I/R injury.

**Results:**

Allicin protected renal I/R injury by ameliorating histological injury and decreasing the oxidative stress in renal tissues. Meanwhile, allicin significantly downregulated the expression of Bax and caspase-3, upregulated the expression of Bcl-2 in I/R renal tissues and H/R treated NRK-52E cells.

**Conclusions:**

Allicin may exert anti-apoptotic and antioxidative effects to promote renal function recovery in I/R renal tissues and H/R treated NRK-52E cells. Taken together, allicin may be a potential novel therapy option for future renal injury protection.

## Introduction

Renal ischemia–reperfusion injury (IRI) occurs after haemorrhagic shock or major cardiovascular surgery and renal transplantation, contributing to renal function impairment and other morbidities. It is the most common cause of the renal failure or hypofunction [1]. Reperfusion of previously ischemia renal tissue triggers complex cellular events that lead to renal cells damage and eventual death [2]. Although reperfusion is critical for ischemia tissue survival, evidence suggests that reperfusion itself can cause additional cellular damage [3]. The molecular mechanisms underlying renal IRI are complex and are not well-understood. Therefore, it is of great significance to understand the exact molecular mechanisms of IRI pathogenesis and discover new as well as more effective treatment methods.

Garlic is one of the longest cultivated plants worldwide. Garlic is a part of a monocot genus (also known as the onion genus) of flowering plants. Allicin is a major bioactive ingredient of garlic, with a variety of biological activities. In the ancient and middle centuries, and for a long time in modern times, garlic was considered as a medicine by doctors in various countries. Especially in recent years, scientific research on garlic has achieved good results in the treatment of many diseases. Various studies have shown that the pharmacological effects of garlic are related to its wide spectrum of beneficial effects such as anticancer, anti-atherogenic, anti-hypertensive, anti-inflammatory, nephroprotective and lipid-lowering activities [4–6]. Allicin has also been reported to have antioxidative properties, protecting the brain from ischemia injury [7]. Rajbir et al. [8] reported that the administration of garlic extract might prevent ischemia–reperfusion-induced myocardial injury, probably by inhibiting oxidative stress. In addition, Ma et al. [9] utilized an ischemia/hypoxia model to induce apoptosis in H9c2 cells in which allicin significantly increased cell activity and decreased the rate of apoptosis. However, the effect and possible mechanisms of allicin on renal IRI remain unknown. Therefore, in the current study, we aimed to investigate the effects of allicin in I/R renal tissues and H/R treated NRK-52E cells.

## Materials and methods

### Animals

Male Sprague Dawley (SD) rats were purchased from the Animal Resource Centre, Wannan Medical College (Wuhu, China). Forty-eight male rats with a weight of 250–300 g were used. The rats were housed in a light-controlled (8 a.m./8 p.m.), homoeothermic (20 °C–22 °C) and air-filtered cages, and were allowed free and easy access to both water and food. All surgical and investigational techniques were conducted as per the recommendations of the Institution Animal Care and Use Committee at Wannan Medical College.

### Animal handling and experimental design

The SD rats were first anaesthetized with 2% sodium phenobarbital (50 mg/kg) and then they were kept on an electrical heating pad to keep a consistent rectal temperature of 37 °C–38 °C. All forty-eight male rats were arbitrarily distributed into six groups. Each group contained eight rats. The rats in group A (control group) were the ones without any surgical operation. The rats in group B (sham group) had completely separate right renal vein through the mid-abdominal incision, and 50 mg /kg allicin was injected intraperitoneally. The rats from group C (I/R group) had a median abdominal incision. The right renal artery and vein were isolated from the surrounding tissues by blunt dissection. After abdominal laparotomy, the right kidney was visible and it was harvested. The right renal pedicle was also visible and it was clamped using vascular forceps for the duration of 45 min ischemia. Following this, the vascular forceps were detached to induce 12 h reperfusion, and then the incision was sealed. The animals in group D, E and F also went through the surgeries which were done in rats in group C followed by allicin administration at concentrations of 40, 50 or 60 mg/kg. Afterwards, the right kidney was harvested and used for further investigation as described below. After the kidneys were removed, it was rinsed in cold normal saline, the blood was removed, the filter paper was dried, and weighed accurately, and then fixed using 10% phosphate-buffered formalin or was instantly frozen and preserved at − 80 °C for histological analysis, and the other was used for biochemical assays.

### Histological examination

The fragments of kidney tissues were fixed using 10% neutral-buffered formalin solution. Following this, the tissue was embedded in paraffin and were cut into small sections which were 4 µm thick. Then, the tissue was deparaffinized, rehydrated, and subsequently stained using haematoxylin and eosin for examination. The morphology was evaluated by a qualified kidney pathologist who did not know have any information about the experimental treatments, using standard methodology. A well-known classification (0–4 levels) was used for the histopathological assessment of IRI injury (10).

### Measurements of malondialdehyde (MDA) levels and enzyme activity of superoxide dismutase (SOD)

To measure antioxidant enzyme activity, frozen tissue samples of the ischemia zone were first homogenized and then centrifuged with 3000 × g at 10 min. After centrifugation, the xanthine oxidase method and the thiobarbituric acid (TBA) reaction were used to determine MDA and SOD, respectively. And each assay was conducted three times. The levels of MDA and the enzyme activity of SOD in renal tissue samples were determined with the help of a spectrometer using commercially available assay kits (Jiancheng Biologic Project Co, Nanjing, China). The MDA levels and the SOD activity were expressed as nmol/mg protein and U/ mg protein, respectively.

### TUNEL assays

To determine the cell apoptosis that was induced by ischemia, the in situ apoptosis detection kit (Promega, USA) was used and the end-deoxynucleotidyl transferase dUTP nick end labelling (TUNEL) was analyzed according to the information provided by the supplier. The normal cell nuclei were bluish-green in colour while the apoptotic cell nuclei were of various shades of brown. To determine the average number of apoptotic cells/100 cells, 5 high-power views were selected for each slide. The apoptotic index (AI) was expressed in form of percentage (%).

### Immunohistochemistry

Five-μm tissue sections were dewaxed and treated with gradient alcohol and water combined with streptavidin immuneoperoxidase method. Briefly, the tissue slices underwent antigen retrieval by microwave oven treatment in 0.01 mol/L of citrate buffer (pH 6.0) for 10 min. The sections were then incubated with 10% normal serum for 30 min, and then incubated overnight with properly diluted (1:100) primary antibodies such as Bax, Caspase-3, and Bcl-2 (Beyotime Inc.China) at 4 °C. Next day, the sections were further incubated for 15 min with either biotinylated anti-mice or anti-rabbit immunoglobulins at room temperature (37 °C). Using a defined rectangular field area at 200 × magnification, 4 slide fields were arbitrarily observed. Further, in each field, Bax, Caspase-3, and Bcl-2-positive cells were counted.

### Cell culture and hypoxia/reoxygenation model

NRK-52E cells were purchased from ATCC (American Type Culture Collection, Manassas, VA, USA). Cells were cultured in Dulbecco’s modified Eagle’s medium (DMEM; GIBCO, MA, USA) mixed with 10% fetal bovine serum (FBS) 37 °C in a humidified incubator containing 5% CO2. To mimic H/R (hypoxia/reoxygenation) injury, the cells were kept in an anaerobic chamber filled with 5% CO2 and 95% N2 at 37 °C for 24 h,and then allicin was added at various final concentrations (1, 3 or 5 μM), followed by normoxic (20% O_2_) conditions for 6 h. Based on the above results, cells were assigned into five groups: group A (control group), group B (H/R group), group C, D and E followed by allicin administration at concentrations of 1, 3 or 5 μM in 24 h hypoxia/ 6 h reoxygenation treatments.

### Flow cytometry assay

The rate of apoptosis was measured using the Annexin V-FITC/Propidium Iodide (PI) staining kit (DOJINDO, Japan; AD10). NRK-52E cells were seeded in 6-well plates at a density of 10^6^ cells/mL. The cells were stained with Annexin V-FITC for 10 min in the dark. Then, 5 mg of ml PI was added to each sample for 30 min so that flow cytometry could be performed (BD PharMingen, San Jose, CA, USA). For the statistical analysis, more than 20,000 total cells per sample were measured.

### Western blot analysis

RIPA buffer along with the phenylmethanesulfonyl fluoride (Beyotime, Nanjing, China) inhibitor was used to extract proteins from kidney tissues and NRK-52E cells. The protein concentration was determined using a BCA protein assay kit (Beyotime, Nanjing, China). 10% SDS-PAGE gels were used and protein samples with a concentration of 40 µg were loaded into each well of the gel. Nitrocellulose membranes (Millipore, Billerica, USA) were used to transfer proteins. Once the transfer was complete, the membranes were incubated for blocking using 5% skim milk made in TBST buffer for 2 h. Next, they were incubated overnight with various primary antibodies at 4 °C such as Bax (1:500 dilutions; sc-493; Santa Cruz Biotechnology), Bcl-2 (1:1000; sc-7382; Santa Cruz Biotechnology) and caspase-3 (1:1000; sc7148; Santa Cruz Biotechnology). Next day, they were incubated at room temperature for 1.5 h with the secondary antibody coupled with horseradish peroxidase (1:1000, Santa Cruz Biotechnology, CA, USA). The protein was detected using ECL reagents (Millipore, Billerica, USA).

### Reverse transcription-polymerase chain reaction (RT-PCR)

Trizol reagent from Invitrogen Technologies (CA, USA) was used to isolate the total RNA from NRK-52E cells. Equivalent quantities of RNA were determined by spectrophotometry and RNA gels and were intended for first-strand cDNA synthesis with Superscript II (Invitrogen Life Technologies) in a 20-µl reaction. Further, the obtained cDNA product (volume used: 1 µl) was used for reverse transcription-PCR (RT-PCR) with the enzyme Taq polymerase (Boehringer Mannheim GmbH, Mannheim, Germany). Using a Light Cycler system (Roche Diagnostics, Mannheim, Germany) quantitative RT-PCR was performed. To detect PCR products, 2X SYBR Premix Ex Taq (Takara Bio Inc., Shiga, Japan) was used. The primer sequences for Bax, Bcl-2 and caspase-3 were as follows: Bax forward, 5'-TGAACTGGACAACAACATGGAG-3', and reverse, 5'-AGCAAAGTAGAAAAGGGCAACC-3'; Bcl-2 forward, 5'-TTAGCGGACCCGGCAAGTGA-3', and reverse, 5'-ATAGTGTCGACATGTCCTCA-3'; caspase-3 forward, 5'-TGGACTGCGGTATTGAGACA-3', and reverse, 5'-GCGCAAAGTGACTGGATGAA-3'. As a housekeeping gene GAPDH was used. The primer sequences for GAPDH: Forward, 5'-ACAGCAACAGGGTGGTGGAC-3' and reverse, 5'-TTTGAGGGTGCAGCGAACTT-3'. The obtained results were mentioned as a ratio to GAPDH mRNA. The cycle details for qPCR are: 40 cycles at 94 °C for 30 s, subsequently at 56 °C for 30 s and at 72 °C for 25 s. To analyse comparative modifications in gene expression, the comparative cycle threshold (Ct) method which is also known as 2^∆∆Ct^ was implemented.

### Statistical analysis

Data were analysed using SPSS (Version 20.0, IL, USA). The Student’s *t* test was used to compare the means of different groups. Statistical significance was achieved with a *p* < 0.05.

## Results

### Allicin alleviates histopathological damage and reduces levels of oxidative stress after renal I/R in rats

**Fig. 1:**
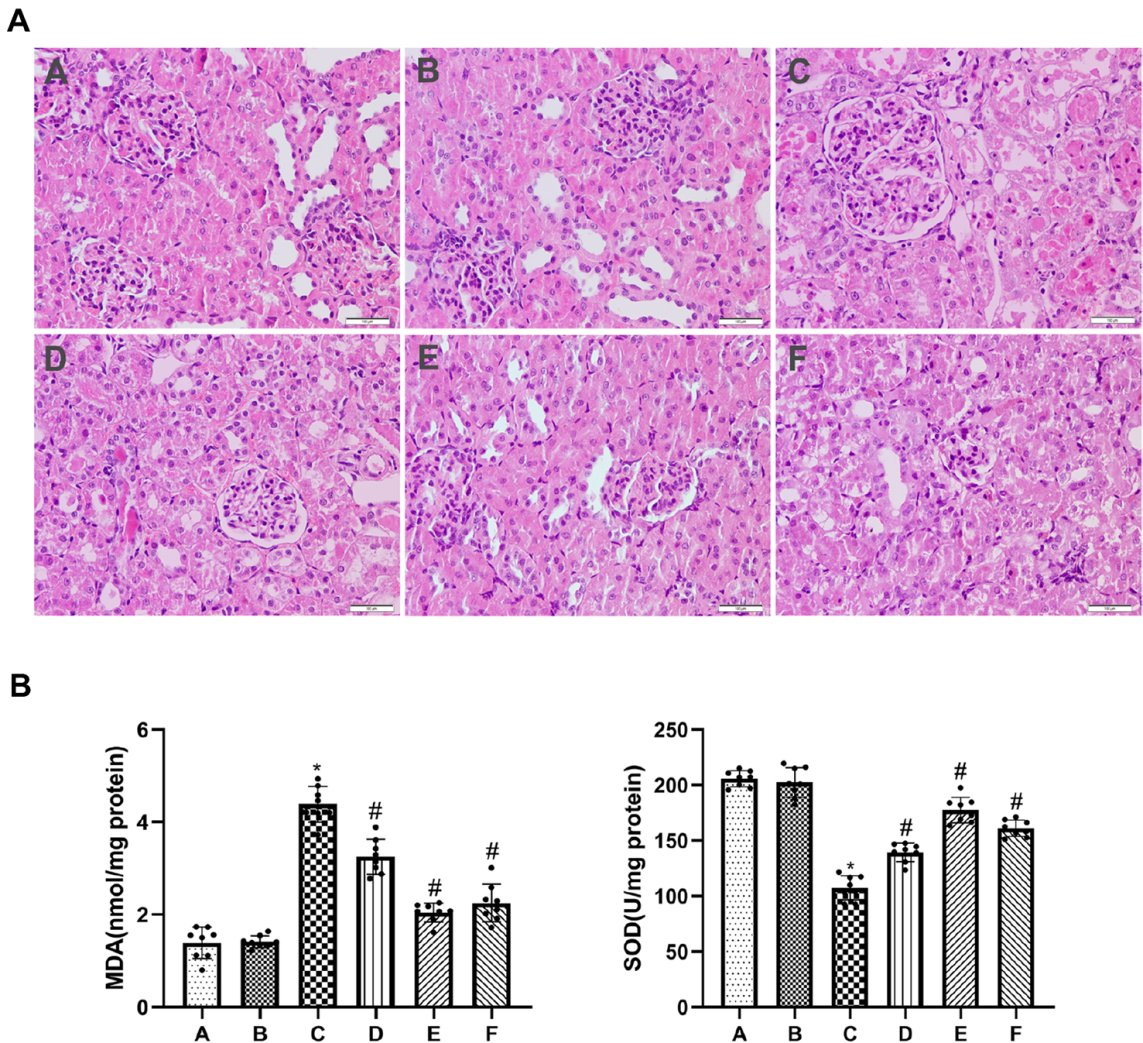
Allicin alleviates histopathological damage and reduces levels of oxidative stress after renal I/R in rats. **A** Hematoxylin and eosin (H&E, × 200) staining was measured to evaluate renal tubule injury in renal tissues. **B** MDA content and SOD activity were measured to evaluate the level of oxidative stress. (**A**) control group, (**B**) sham group, (**C**) I/R group, (**D**) I/R + 40 mg/kg allicin group, (**E**) I/R + 50 mg/kg allicin group and (**F**) I/R + 60 mg/kg allicin group. *p < 0.05 vs. group A and B; ^#^*p* < 0.05 vs. group C

HE staining showed that there were no obvious histopathological changes in the renal tissue of rats in groups A and B. Renal I/R resulted in significant damage to renal function, as evidenced by the dilatation and congestion of renal tubules as well as the swelling and necrosis of renal tubular epithelial cells. However, treatment with allicin reduced severe renal damage in groups D, E and F compared with that in group C (Fig. 1A). To evaluate the levels of oxidative stress in renal I/R, MDA levels and activity of SOD enzyme were determined in renal tissue. Compared with groups A and B, the MDA content was significantly higher, and the SOD activity was substantially lower in group C. Allicin ameliorated the increase in MDA content and the reduction in SOD activity induced by renal IRI in groups D, E and F (Fig. 1B).

### Allicin inhibits the apoptosis of renal tubular epithelial cells after renal I/R in rats

**Fig. 2:**
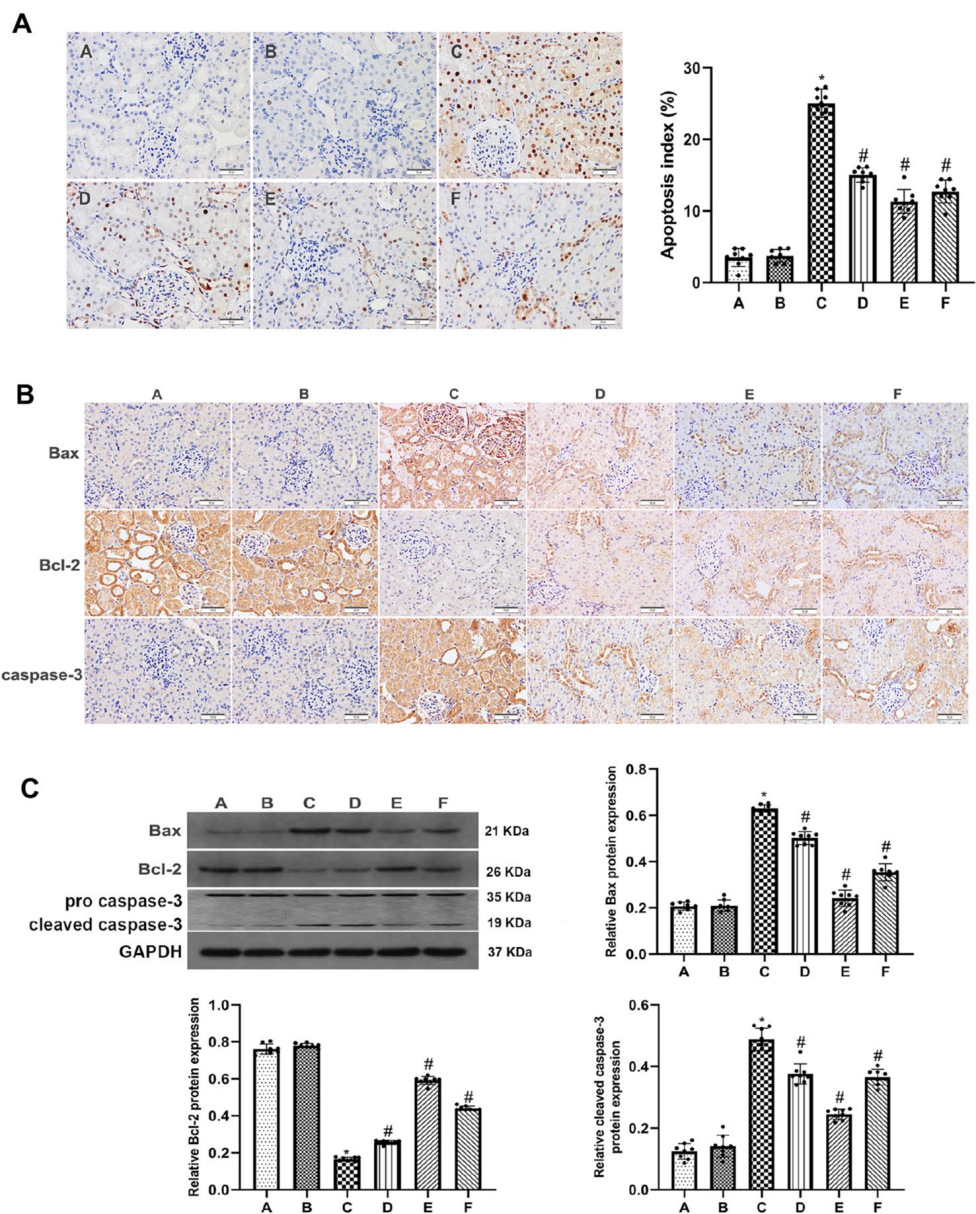
Allicin inhibits the apoptosis of renal tubular epithelial cells after renal I/R in rats. **A** Apoptosis index (AI) in the 6 groups (magnification, × 200). **B** Immunohistochemistry was performed to measure the expression of Bax, caspase-3 and Bcl-2 (magnification, × 200). **C** The protein expression of Bax, cleaved caspase-3 and Bcl-2 was detected by western blot. GAPDH was as a loading control. (**A**) control group, (**B**) sham group, (**C**) I/R group, (**D**) I/R + 40 mg/kg allicin group, (**E**) I/R + 50 mg/kg allicin group and (**F**) I/R + 60 mg/kg allicin group. *p < 0.05 vs. group A and B; ^#^*p* < 0.05 vs. group C

To observe apoptosis and determine the apoptotic index (AI), the expression of Bax, Bcl-2 and caspase-3 in renal tubular epithelial cells was measured by TUNEL assays, immunohistochemistry, western blot analysis. There were significantly more TUNEL-positive cells in group C than in groups A or B. Treatment with allicin at a dose of 50 mg /kg had an ideal therapeutic effect on the damaged kidney, evidenced by a significant reduction of TUNEL-positive cells (Fig. 2A). In group C, the expression of caspase-3 and Bax was markedly higher, while the expression of Bcl-2 was significantly lower. However, treatment with allicin decreased the expression of caspase-3 and Bax and elevated the expression of Bcl-2 in groups D, E and F. The results indicated that allicin was most effective at attenuating this dysfunction at a dose of 50 mg /kg (Fig. 2B, C).

### Allicin inhibits the apoptosis of renal tubular epithelial cells induced by H/R in NRK-52E cells

**Fig. 3:**
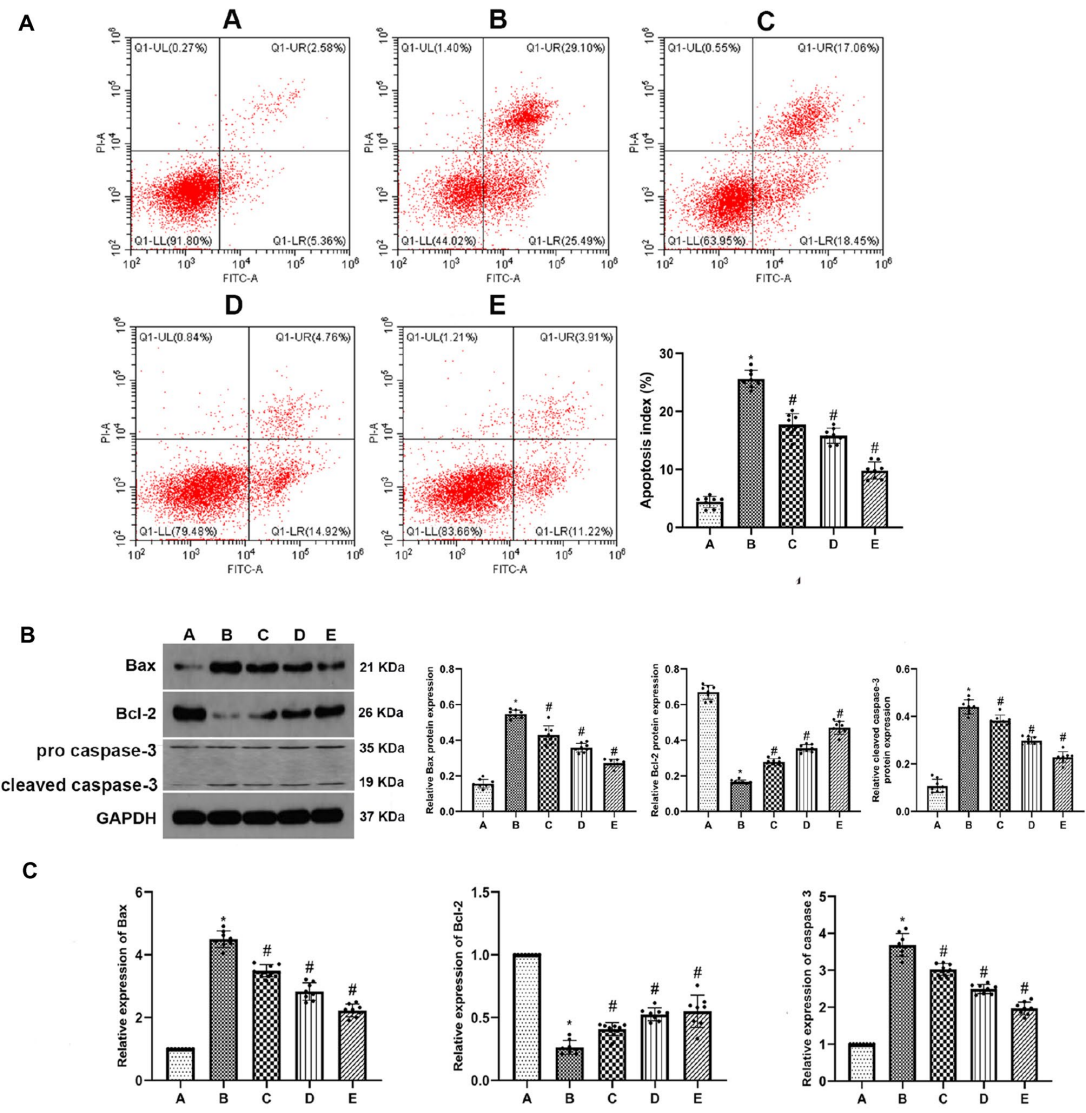
Allicin inhibits the apoptosis of renal tubular epithelial cells induced by H/R in NRK-52E cells. **A** Cell apoptosis of NRK-52E cells was examined by flow cytometry. **B** The protein expression of Bax, cleaved caspase-3 and Bcl-2 was detected by western blot. GAPDH was as a loading control. **C** The mRNA expression of Bax, caspase-3 and Bcl-2 was evaluated by qRT-PCR analysis. (**A**) control group, (**B**) H/R group, (**C**) H/R group + 1 μM allicin group, (**D**) H/R + 3 μM allicin group, (**E**) H/R + 5 μM allicin group. *p < 0.05 vs. group A; ^#^*p* < 0.05 vs. group B

The rate of apoptosis was determined by flow cytometry after staining the NRK-52E cells with Annexin V-FITC/PI. In the group B, cell apoptosis significantly increased compared with group A. Cell apoptosis in the allicin-treated groups significantly decreased compared with group B (Fig. 3A). Furthermore, the expression of Bax, Bcl-2 and caspase-3 in renal tubular epithelial cells was measured by western blot and RT-PCR analysis. The results showed that the expression of caspase-3 and Bax increased markedly, while Bcl-2 showed decreased expression in group B compared to that in group A. However, treatment with allicin decreased the expression of caspase-3 and Bax and increased the expression of Bcl-2 in groups C, D and E. (Fig. 3B, C).

## Discussion

During surgeries such as renal transplantation, nephron-sparing surgery, and cardiovascular surgery, transient ischemia and consequent reperfusion happen generally in the kidney. But this type of decrease or disruption of renal perfusion and consequent reflow frequently lead to renal injuries. When the blood supply to an ischemia body part is re-established, the influx of oxygen occurs and energies the extreme production of reactive oxygen species (ROS). Thus it creates paradoxical reperfusion injury (oxygen paradox) as these ROS can alter lipids. proteins, nucleic acids and glucose in tissues and cells and leads to cell dysfunction and cell death [11]. Necrotic cell death leads to activation of various inflammatory signalling pathways, and thus causes severe secondary tissue damage [12]. With the increasing frequency of IRI, its pathogenesis and prevention are attracting increasing attention.

Allicin, an active ingredient of garlic, has numerous medicinal benefits, and the plant has been necessary in everyday life from the past until the present day. Allicin is responsible for the effect of garlic on almost every part of the human body. The anticholesterolemic and antilipidaemic action of garlic has been reported in rabbits and rats, and the antihypertensive action of garlic has been shown in rats [13, 14]. Allicin has been tested in in vitro studies for anti-tumour, anti-inflammatory, antioxidative and anti-microbial activities [15, 16]. Chen et al. [17] established that allicin could protect human umbilical vein endothelial cells (HUVECs) from H_2_O_2_-induced cell apoptosis by inhibiting oxidative stress. Kong et al. [7] reported neuroprotective beneficial effects of allicin on ischemia–reperfusion brain injury, and this protection was likely mediated by a decreased production of free radicals and inflammatory factors. Zhu et al. [18] showed that allicin can protect against IRI of the spinal cord. Nevertheless, it has not previously been demonstrated whether allicin protects tissue against IRI. The present novel data have demonstrated that I/R severely damages the renal function and various doses of allicin ameliorated histological injury to different extents. The ideal effect was induced following the administration of 50 mg /kg in experimental rats.

Oxidative stress has long been considered a key factor in the pathogenesis of IRI for a long time [19]. Oxidative stress is triggered initially by dysfunction of the mitochondrial respiratory chain in the ischemia phase and is magnified in the reperfusion phase, causing cell death by directly impairing DNA, proteins and lipids [20]. Normally, the ROS produced by metabolic processes can be eliminated by endogenous antioxidant enzymes such as SOD [21], catalysing the dismutation of the superoxide radical to hydrogen peroxide. Excessive ROS production and reduction of antioxidant capacity results in the deterioration of renal IRI [22]. In addition, MDA, an important product of lipid oxidation, is often used to study the effect of oxidative stress on cell injury. It has been reported that lowering the elevation of MDA levels and inhibiting SOD activity has protective effects on cardiac IRI [23, 24]. Our study showed that allicin can significantly reduce MDA level elevation and decrease SOD activity after renal I/R. Therefore, we believe that allicin treatment might reduce oxidative stress, increase antioxidant capacity, and decrease subsequent renal injury.

Previous studies have shown that apoptosis is closely related to the pathological process of renal IRI [25]. As an anti-apoptotic protein, Bcl-2 can not only prevent the formation of lipid peroxides but can also depress the release of endoplasmic reticulum Ca2 + and free radical production [26]. Bax, is an endogenous antagonist of Bcl-2. It causes inhibition of the protein by binding directly to the related protein homologues, thereby promoting cell apoptosis [27]. Under healthy condition, the Bcl-2 and Bax expressions are generally well-balanced [28]. However, in the current study, we have reported that IRI activated renal cell apoptosis by augmenting Bax protein expression and by decreasing Bcl-2 protein expression. Activation of caspases is a significant biochemical feature of cell apoptosis [29]. The caspase family of cysteine proteases is involved in the initiation and execution of mammalian apoptosis procedures. Caspase-3 is formed from 32 kDa zymogen and cleaved to the active 17 kDa subunit through death ligand and mitochondrial pathways [30]. This zymogen is a key effector of caspase that initiates degradation of the cell in the final stages of apoptosis [31]. Excessive ROS activates both mitochondrial and endoplasmic reticulum stress pathways. When the mitochondria were damaged, the increase of Bax and the decrease of Bcl-2 changed the ratio of Bax to Bcl-2 in the mitochondrial outer membrane and promoted the release of cytochrome c. The release of cytochrome c activates the apoptosis effector protein caspase-3, and ultimately induces mitochondrial-dependent apoptosis [32]. Our study has demonstrated that allicin treatment significantly alleviated cell apoptosis, as shown by decreased the expression of Bax and caspase-3, while increased the expression of Bcl-2.

In conclusion, this study demonstrated for the first time that the administration of allicin exerted anti-apoptotic and antioxidative effects to promote renal function recovery in I/R renal tissues and H/R treated NRK-52E cells. Although the protective mechanism of allicin has not been fully elucidated, allicin can represent an easy-to-use, safe, low-cost, effective, and novel treatment option for future renal injury protection.
